# Effect of 30 years of road traffic abandonment on epiphytic moss diversity

**DOI:** 10.1007/s10661-014-4056-3

**Published:** 2014-09-27

**Authors:** Vítězslav Plášek, Arkadiusz Nowak, Marcin Nobis, Grzegorz Kusza, Katarzyna Kochanowska

**Affiliations:** 1 Department of Biology and Ecology, University of Ostrava, 710 00 Ostrava, Czech Republic; 2 Department of Biosystematics, Laboratory of Geobotany and Plant Conservation, Opole University, 45-052 Opole, Poland; 3 Department of Plant Taxonomy, Phytogeography and Herbarium, Institute of Botany, Jagiellonian University, 31-501 Kraków, Poland; 4 Department of Land Protection, Opole University, 45-052 Opole, Poland; 5 Laboratory of Biodiversity and Ecology, Institute of Biology, Tomsk State University, 36 Lenin Prospekt, Tomsk, 634050 Russia

**Keywords:** Moss monitoring, Road salting, Tree conservation, Epiphytes, *Tilia cordata*, *Orthotrichum*, Poland

## Abstract

Road traffic emits a cocktail of pollutants that can influence the vegetation and plant diversity in neighboring areas. However, the recovery potential of bryophytes after traffic abandonment is still little explored. In addition, the effects of the main pollutants of road verges, such as metals and salinity, on moss flora need to be investigated. In our study, we compared the moss richness and diversity in two closely related veteran tree allees of high conservation importance. The allees in Gryżów and Lubrza, Poland, were chosen because of their similarity in age, geographical location, type of surrounding areas, and tree species. The only difference was that the trees in Gryżów had not been exposed to direct road pollution for almost 30 years. The moss richness and diversity differed significantly between the sites. Altogether, 20 moss species were recorded on 229 trees, 17 species in Gryżów (abandoned road), and 13 in Lubrza (busy road). We found considerable differences between moss cover on the road-facing and opposite sides of tree trunks. In Lubrza, mosses on the road-facing side were very scarce. The moss cover in Gryżów was highly balanced between trunk sides as well as among trunk heights. Typical epiphytic species such as *Bryum moravicum*, *Dicranoweisia cirrata*, *Leskea polycarpa*, and *Orthodicranum tauricum* preferred the Gryżów tree stands, where they were present in numbers almost twice as high as that at Lubrza. The study shows that constructing a bypass road could be an effective conservation measure for veteran tree protection with their epiphytic moss flora.

## Introduction

Motor vehicles emit a complex mixture of pollutants. The most important airborne toxic substances from road transport are nitrogen oxides (NOx), ammonia (NH_3_), nitrous acid (HONO), heavy metals (e.g., Zn, Pb, Ni, and Cd), volatile organic compounds (VOCs), polycyclic aromatic hydrocarbons (PAHs), and others (e.g., Bignal et al. 2007, 2008). In addition, road salting could have a strong phytotoxic effect on road verge vegetation (Thompson et al. 1986). Because of this, in recent years, much research has focused on the ecological effect of traffic pollution on vegetation and sites of conservation interest (Angold 1997; Spellerberg 1998; Forman 2000; Aničić et al. 2007; Kłos et al. 2012). One such type of site could be a legally protected old tree avenue. In Opolskie Province (SW Poland), almost 35 % of provincial tree monuments established on the basis of the Nature Conservation Act (Ustawa 2004) are located within road verges. It is known from experimental research and field surveys that road traffic significantly influences the diversity of plants, especially trees. Many scientific studies have made evident that road pollution has a significant effect on plant growth, physiology, enzyme activity, chemistry, senescence, leaf/needle surface wax degradation, and plant-insect interactions (Bignal et al. 2004). Several of those studies have documented impacts on the health of trees growing in rows along roads or in stands of forest trees (e.g., Bignal et al. 2008). There are also studies focused on the bryophyte response to high pollutant concentrations in urbanized areas, for example, smoke and sulfur dioxide (Gilbert 1968; Larsen et al. 2007; Bignal et al. 2008). However, little is known about the direct influence of toxic substances on epiphytic moss diversity and thresholds of toxicity of different substances in relation to specific moss species. Morgan et al. (1992) found that exposure to 35 ppb nitrogen dioxide (NO_2_) or nitric oxide (NO) over 21 days affected nitrate reductase activity and/or oxygen evolution in four bryophyte species. Bell et al. (1992) observed stimulated and then reduced growth in *Polytrichum formosum* exposed to 60 ppb NO_2_ for 37 weeks. This increase in growth is explained as a result of fertilization from the increased deposition of NOx, HONO, or NH_3_. A direct negative effect on *Sphagnum* species has been documented by Potter et al. (1996).

**Fig. 1:**
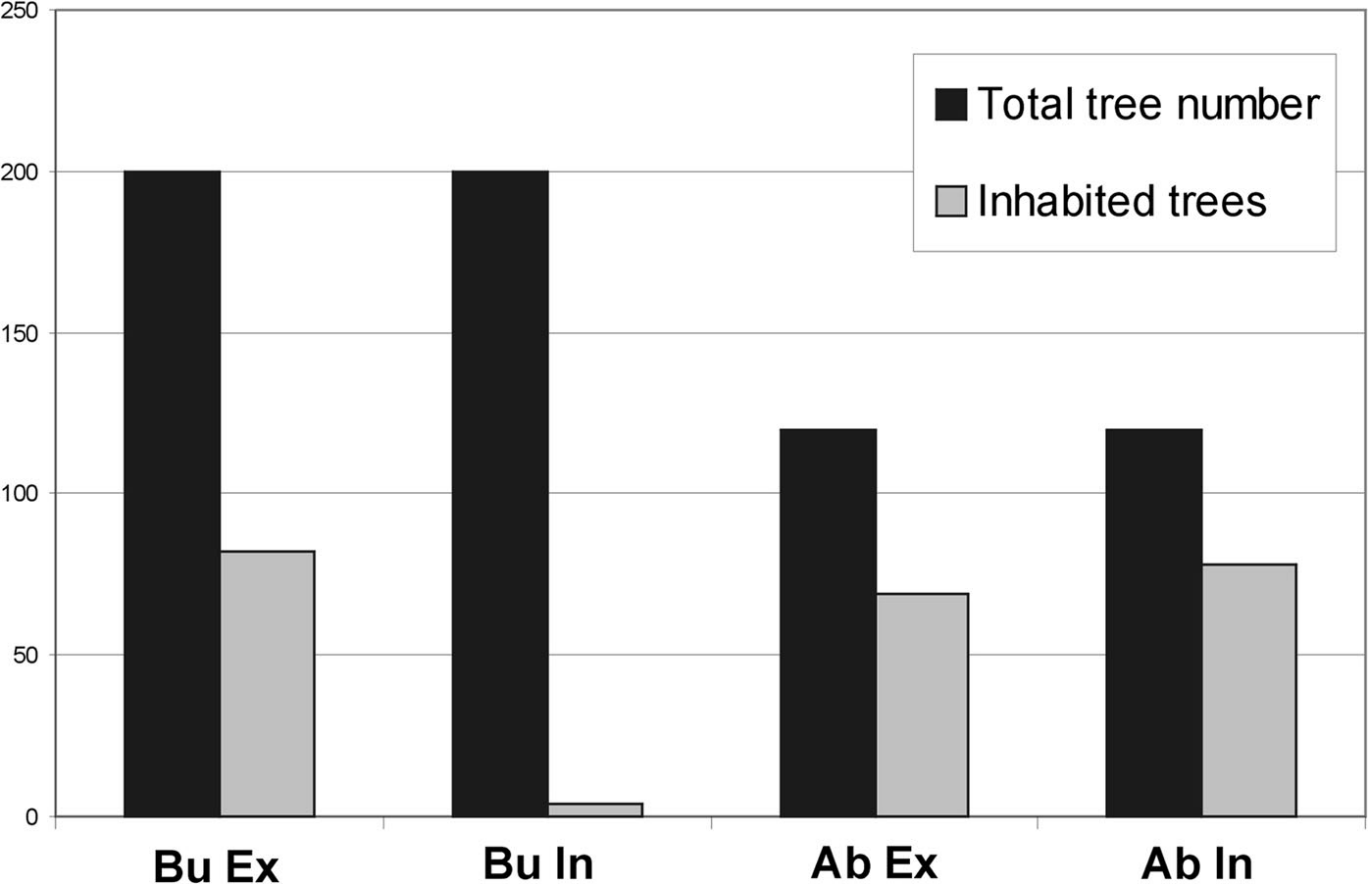
Bryophyte occupancy of moss species on road-facing side (*In*) and opposite side of trunks (*Ex*). *Bu* busy road in Lubrza, *Ab* abandoned road in Gryżów

Many surveys that have examined bioaccumulation and biomonitoring in mosses have shown that some bryophytes are well adapted and resistant to changing environmental conditions and high pollution. Many of the studies used mosses exposed in nylon bags as heavy metal traps. This useful technique has been common for more than 40 years in many countries (e.g., Goodman and Roberts 1971; Castello 1996; Samecka-Cymerman and Kempers 2007; Tretiach et al. 2007; Aboal et al. 2008a, b; Kosior et al. 2010) and recently gained a standardized methodology and monitoring protocol (Ares et al. 2012). The use of terrestrial mosses as bioaccumulators of toxic airborne contaminants such as As, Cd, Hg, Ni, or Pb provide effective and relatively affordable alternatives for monitoring the pollutants compared to that by standard physicochemical techniques (e.g., Kongtip et al. 2006; Yang and Omaye 2009).

The epiphytic habitat is mainly characterized by dryness due to the vertical position of the substratum, high light intensity, and sharp alternation of dry and humid periods. Epiphytes are organisms that grow on a living plant for support and are not parasites. Epiphytes usually obtain water and nutrients from fog, dew, or rainwater. Water availability for epiphytic plants is irregular, and plants tend to endure drought stress between precipitation events. Dry periods are particularly crucial, especially for juvenile plants, which are very sensitive to drying. Bryophytes are of ecological importance, and the effects on this component of the flora could have wider implications for the habitat with which they are associated. The invertebrate fauna among bryophytes is considerably diverse and consists of many species with different life strategies and cycles, such as insects, nematodes, rotifers, tardigrades, and annelids, and is likely to be the most responsible for nutrient and detrital material cycling within the moss communities, thus, considerably influencing the habitat of bryophytes (Merrifield and Ingham 1998; Glime 2013).

It is also worth mentioning the significance of old, mature trees for bryophytes and their communities. Many authors have highlighted the importance of old, mature, and veteran trees in supporting moss diversity in forests (e.g., Király and Ódor 2010; Zechmeister et al. 2003; Ódor et al. 2013) as well as in cultural landscapes (e.g., Orłowski and Nowak 2007). The tree hedgerows or avenues are often the mid-field refuges for many vascular plants or moss species (Burel and Baudry 1990; MacDonald and Johnson 2000). Linear woodlots have been recognized as an important ecological corridor in the migration of species typical for woodlands (Le Cour et al. 2002). This issue is also relevant to monumental trees under legal protection (Orłowski and Nowak 2007; Jim 2004).

Despite the extensive surveys focused on the conservation value of epiphytic mosses and the influence of airborne pollutants on moss flora, little is known about the response of bryophyte diversity and abundance to long-term road pollution alleviation.

The present study assesses the effect of road traffic abandonment for 30 years on moss species diversity and distribution. Further, the role of mature tree avenues in moss conservation is discussed. We also try to determine the main causes of moss decline on tree bark exposed to pollution from road transport.

## Material and methods

### Study area

The study area is located in Opole Province in the southwestern part of Poland between 16° 53′ 40″–18° 41′ 50″ E and 49° 58′ 05″–51° 18′ 20″ N. The region is characterized by agricultural lands, which cover almost 63 % of the province. Forests occupy about 26 %, communication areas 3.8 %, open waters 2.2 %, and urban, residential, and industrial areas about 5 % (Nowak and Nowak 2004a). The two tree avenues with epiphytic mosses are situated in the southern part of the province on Głubczyce Plateau, ca. 12 km apart: Lubrza, 17° 37′ 41″ E, 50° 20′ 44″ N, altitude 254 m above sea level (a.s.l.); Gryżów, 17° 29′ 23″ E, 50° 24′ 54″ N, altitude 273 m a.s.l. Each of the tree hedgerow is consists of two parallel rows of trees on the sides of the road. In Lubrza, the rows consist of 200 trees of *Tilia cordata* (100 on each side) ca. 220 years old; in Gryżów, the rows consist of 120 trees of *Tilia cordata* (75 in the southern and 45 in the northern row) of the same age. The tree hedgerows had been planted as was common in Silesia alongside the main provincial road connecting the cities of Nysa, Prudnik, and Opole. Because of their ornamental value and traditional and historical meaning, the tree plots have been legally preserved according to a provincial ordinance from 1957 as Provincial Monument numbers 234 and 274 (Ordinance 1957). They were designated as national monuments according to the Polish law of nature conservation (Ustawa 2004).

From the beginning, the trees were exposed to very similar pollution. The road traffic was the same during the nineteenth and twentienth centuries because the trees were planted alongside the same type of road (district road), and the human population density in the surrounding area was very similar (rural areas). Recently (2000–2010), according to the Provincial Road Headquarters, the average daily road traffic is 6,447 vehicles (road section DK41 Nysa-Prudnik alongside Gryżów allee) and 6,683 (section DK40 Lubrza-Prudnik via Lubrza allee) (Report 2010). The tree plots are also quite similar in their neighboring agricultural lands, lack of forest plots in close vicinity, and geographical orientation (generally a west–east direction of the tree rows). During the winter, the road in Lubrza is deiced. The salt mixture is mostly NaCl with minor amounts of CaCl_2_, MgCl_2_, and KCl. In the winter of 2012/2013, the average amount of deicing salt used in Opole Province on district roads was ca. 250 kg per kilometer. As was reported by Blomqvist and Johansson (1999), up to 90 % of salt used for deicing is spread as an aerosol and deposited on the ground 2–35 m from the road.

Because of the poor health of trees and conservation recommendations, in 1984, the road in Gryżów was closed to public traffic and a bypass section was built outside ca. 18–22 to 30–35 m away from the tree rows (distance from the middle line of new road to both tree rows). Thus, for 30 years, the toxic pollutants and salt burdens have been significantly diminished in Gryżów.

### Sampling design and data analyses

We chose 200 trees (75 % of the trees) in busy road (Lubrza) and 120 (100 % of the trees) in abandoned road (Gryżów) to collect moss data disregarding the tree girths. All specimens belonged to *Tilia cordata* with the exception of one individual of *Acer platanoides*, which was excluded from the analysis. We sampled each tree trunk on both sides (the road-facing side and opposite side) at three different heights: 20, 120, and 220 cm. At each height, we recorded all moss species on a sample plot of 40 cm^2^. Percentage moss cover was recorded. The moss species were determined using recent keys focused on epiphytic bryophytes (e.g., Plášek 2012) or general keys for identifying mosses (e.g., Frey et al. 2006). Species nomenclature mainly followed Ochyra et al. (2003), with modifications by Sawicki et al. (2010), Plášek and Sawicki (2010), and Plášek et al. (2011). Plant material collected during field studies was housed at the University of Ostrava herbarium. The study sites were surveyed in October 2013.

The moss species were classified as obligatory epiphytes and facultative epiphytes according to Szövényi et al. (2004). To find the synanthropodynamic state of moss flora on the research tree plots, we characterized moss species based on their frequencies of occurrence on anthropogenic habitats. We defined three groups: (1) species inhabiting only natural habitats (e.g., mainly forests and rock outcrops), (2) species occurring on natural as well as man-made habitats (so called facultative anthropophytes), and (3) typical anthropophytes, with all or almost all of the populations in southern Poland and northern Czech Republic on artificial substrates.

### Analyses of chemical–physical compounds in soil and bark

In each tree line, we collected nine soil and bark samples to determine the main contaminants. The soil samples were taken from the 0–30-cm depth from both sides of 18 trees (nine in Lubrza and nine in Gryżów), in the closest vicinity of the trunk. Accordingly, bark samples were taken from 18 trees at 1-m height, equally on both sides of trunks. The laboratory studies comprised physicochemical analyses of the soil samples and plant material (bark). Initially, the soil samples were dried at room temperature, then sieved with a 2-mm mesh, and homogenized. Bark samples were ground and homogenized. The physical and chemical properties of samples were assessed using the following standard methods: pH, potentiometric method with a glass electrode and a sample/water/1 N KCl ratio of 1:2.5 (soil) and 1:10 (bark); electrolytic conductivity, conductometric method; total carbon content, multi N/C Analytikjena HT1300; heavy metal content (Cu, Zn, Pb, Cd), atomic absorption spectrometry (AAS; ICE 3500, Thermo Scientific) after previous digestion of soil samples with aqua regia; Na, K, Li, and Ca, AAS with flame photometer BWB XP (BWB Technologies UK Ltd); and mercury, AMA 254 spectrophotometer (Czerniawska-Kusza et al. 2004).

### Statistical analyses

We used the Shapiro–Wilk test to test the null hypothesis that the samples came from a normally distributed population. A Wilcoxon signed rank non-parametric test for related samples was used to compare the moss abundances on the road-facing and opposite sides of the trees. For this comparison, the individual species cover was summed for each 40 cm^2^ plot. To find the distribution pattern of mosses on tree trunks, we compared the plots at 0.2, 1.2, and 2.2 m on every trunk using the non-parametric Kruskal–Wallis one-way analysis of variance. To find out the differences between the moss cover of the two avenues, we compared them separately for each side and every height (0.2, 1.2, and 2.2 m). To test the null hypothesis that there was no difference, we used a non-parametric Mann–Whitney *U* test.

The relationships between the sample groups were explored using ordination techniques. A preliminary indirect DCA analysis was launched, which revealed the intermediate gradient length along the ordination axes (3.3 value). This allowed us to implement principal component analysis to find the differences in plot species structure (ter Braak and Šmilauer 2002). The relationship between the species composition and environmental variables (salinity, pH, toxic metals) was further explored by redundancy analysis. In these ordinations, only trees with epiphytes were included (229 samples). Cover values were ln transformed before the performance. The effect of explanatory variables was tested by F-statistics via Monte-Carlo simulation with 499 permutations. The accepted significance level was 0.05. The measured and derived explanatory variables used for RDA are listed in Table 1. For the ordinations, CANOCO for Windows 4.5 was used (ter Braak and Šmilauer 2002; Leps and Šmilauer 2003).

**Table 1:** Explained variance of the explanatory variables used in the redundancy analysis (RDA)

Variable	Variance (%)	*p* value	*F* value
Bark			
Sodium (Na)	4	0.002	14.95
Mercury (Hg)	3	0.078	2.98
Lithium (Li)	6	0.192	1.59
Cadmium (Cd)	2	0.27	1.27
Lead (Pb)	1	0.258	1.26
Potassium (K)	5	0.262	1.11
Electroconductometry (EC)	8	0.412	0.8
Calcium (Ca)	7	0.558	0.6
pH	9	0.762	0.38
Soil			
pH	11	0.002	11.69
Lithium (Li)	8	0.038	3.61
Zinc (Zn)	2	0.04	3.11
Potassium (K)	7	0.122	1.91
Electroconductometry (EC)	10	0.256	1.34
Cadmium (Cd)	4	0.46	0.77
Copper (Cu)	1	0.364	0.92
Lead (Pb)	3	0.348	0.92
Mercury (Hg)	5	0.438	0.72
Calcium (Ca)	9	0.908	0.24
Sodium (Na)	6	0.986	0.14

## Results

**Fig. 2:**
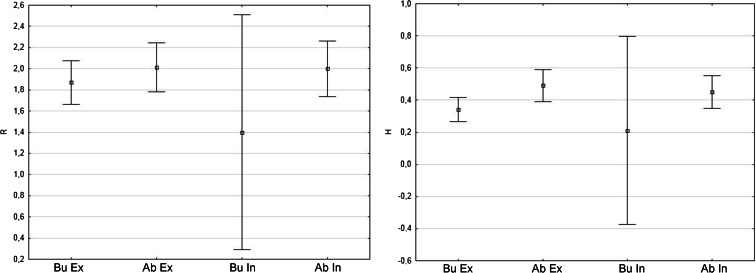
Diversity and species richness of moss communities on road-facing sides (*In*) and external sides (*Ex*) of studied tree trunks in abandoned road (*Ab*; Gryżów) and busy road (*Bu*; Lubrza)

There was apparent difference in moss occupancy between two allees. In Lubrza, mosses colonized ca. 43 % and in Gryżów 66 % of the trees (Fig. 1). All moss species inhabiting the *Tilia cordata* trunks are listed in Table 2 together with their frequencies and abundances. Twenty moss species were recorded on 229 trees: 17 in Gryżów (abandoned road) and 13 in Lubrza (busy road; Fig. 1). The mean number of species per inhabited tree was ca. 2.5 (ranging from 1 to 6). The Shannon diversity index had an average value of approximately 0.42 (ranging from 0.15 to 1.5). The highest moss diversity and richness was noted in abandoned road, with no considerable difference between the sides of trunks (Fig. 2). The most widely distributed moss species, in decreasing frequency, were *Hypnum cupressiforme*, *Ceratodon purpureus*, *Dicranoweisia cirrata*, *Bryum moravicum*, and *Platygyrium repens*. Details of species frequencies on different trunk heights can be found in Table 2.

**Table 2:** Details of species richness and frequencies on different trunk heights in researched allees in abandoned road (Gryżów) and busy road (Lubrza)

Species	Busy road (Lubrza)	Abandoned road (Gryżów)	Total frequency		Frequency at 0.2 m		Frequency at 1.2 m		Frequency at 2.2 m		Average cover on occupied trunks [%]		Minimum cover [%]	Maximum cover [%]
			Bu	Ab	Bu	Ab	Bu	Ab	Bu	Ab	Bu	Ab		
*Amblystegium serpens*	+	+	1	4	1	3	0	1	0	0	3	10.67	2	32
*Barbula unguiculata*	+		2	0	2	0	0	0	0	0	1.25	0	0.5	2
*Brachythecium salebrosum*	+		2	2	2	0	0	0	0	0	16	0	1	17
*Brachythecium velutinum*	+	+	3	11	3	11	0	0	0	0	5.83	4.64	0.5	15
*Bryum moravicum*	+	+	25	1	15	1	5	0	5	0	2.9	0.5	0.5	20
*Ceratodon purpureus*	+	+	52	48	49	48	3	0	0	0	7.32	2.95	0.1	25
*Dicranoweisia cirrata*	+	+	17	109	9	58	4	27	4	24	15.17	6.45	0.5	45
*Grimmia pulvinata*		+	0	1	0	1	0	0	0	0	0	1	1	1
*Hypnum cupressiforme*	+	+	67	153	59	106	7	31	1	16	18.91	7.06	0.3	81
*Leskea polycarpa*		+	0	1	0	1	0	0	0	0	0	2	2	2
*Orthodicranum tauricum*	+	+	1	1	1	1	0	0	0	0	0.5	3	0.5	3
*Orthotrichum affine*	+	+	9	10	7	9	2	1	0	0	1.07	2.06	0.5	7
*Orthotrichum anomalum*		+	0	4	0	2	0	1	0	1	0	3	1	3
*Orthotrichum diaphanum*	+	+	1	3	1	3	0	0	0	0	0.2	2	0.2	3
*Orthotrichum pumilum*	+	+	2	3	1	3	1	0	0	0	1	6	1	8
*Platygyrium repens*		+	0	27	0	16	0	7	0	4	0	4.53	0.5	23
*Pterigynandrum filiforme*		+	0	6	0	5	0	1	0	0	0	1.4	1	2
*Pylaisia polyantha*	+		2	0	2	0	0	0	0	0	51.5	0	43	60
*Tortula muralis*		+	0	2	0	1	0	0	0	0	0	1	1	1
*Tortula papillosa*		+	0	1	0	1	0	0	0	0	0	0.5	0.5	0.5

**Fig. 3:**
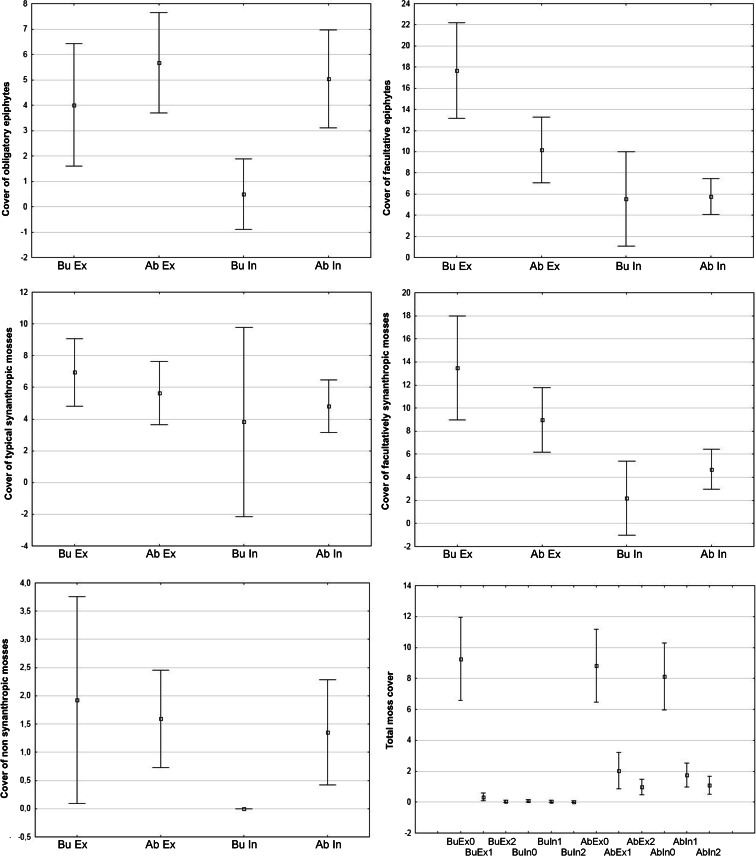
Percentage cover of obligatory and facultative epiphytes, typical and facultative synanthropic mosses, cover of non-synanthropic mosses, and total moss cover on researched plots. *In* internal, road-facing trunk side, *Ex* external tree trunk side, *Cov* summarized moss species cover in researched plot, *Ab* abandoned road (Gryżów), *Bu* busy road (Lubrza), 0–0.2, 1–1.2, and 2–2.2 m

The comparisons of species cover, especially within the functional groups, revealed considerable differences between the tree avenues, as well as the sides of trunks in each tree hedgerow. Total moss cover was highest on the opposite side of trees from the road in Lubrza and Gryżów at the base of the trunks (ca. 9 % on average). Mosses were also considerably abundant at the base on the road-facing side of *Tilia cordata* trunks in abandoned road in Gryżów (ca. 8 % on average). There was almost no moss cover in busy road in Lubrza on the road-facing side at the base of the trunks, as well as at 1.2 and 2.2 m (only 2 % of trees were inhabited). The only species found were *Barbula unguiculata*, *Bryum moravicum*, *H. cupressiforme*, and *C. purpureus*, all with very low abundances. In contrast, in abandoned road in Gryżów the road-facing side of trunks was frequently inhabited by mosses at the tree base, as well as at 1.2 and 2.2 m (Fig. 3).

The cover of typically synanthropic mosses inhabiting southern Poland and the northern Czech Republic mainly in man-made habitats was almost equal in all research plots, but slightly higher in Lubrza facing away from the road. Greater differences were noted for facultatively synanthropic taxa; however, the highest cover was also recorded at busy road in Lubrza facing away from the road. The abundances of moss species typical of natural habitats were relatively similar with the exception of the road-facing side in Lubrza, where they were close to zero (Fig. 3). It was also evident that the typically epiphytic species, such as *Bryum moravicum*, *D. cirrata*, *Leskea polycarpa*, *Orthodicranum tauricum*, *Orthotrichum affine*, *Orthotrichum diaphanum*, *Orthotrichum pumilum*, *Platygyrium repens*, *Pterigynandrum filiforme*, and *Tortula papillosa*, preferred the Gryżów tree stands, where they were almost twice as abundant as at busy road in Lubrza.

The Wilcoxon test for related samples showed significant differences between moss cover values at 0.2- and 1.2-m heights for both sides of trunks (*p* < 0.05; Table 3). In those cases, moss abundances were considerably lower facing the road. Differences at 2.2 m were insignificant because of the scarcity of data. The same comparison made for Gryżów tree rows revealed different results. Despite higher numbers of related pairs, the moss cover at 1.2 and 2.2 m on both sides of trees was almost the same. A small difference was observed at 0.2-m height.

**Table 3:** The statistical results of comparison in related pairs of moss cover on different heights

	N	T	Z	*p* value
Bu (Lubrza) Ex-In				
Cov0Ex Bu and Cov0In Ab	86	82.00000	7.701276	0.000000
Cov1Ex Bu and Cov1In Bu	14	14.00000	2.416895	0.015654
Cov2Ex Bu and Cov2In Bu	4	3.00000	0.730297	0.465209
Ab (Gryżów) Ex-In				
Cov0Ex Ab and Cov0In Ab	85	1309.000	2.271944	0.023091
Cov1Ex Ab and Cov1In Ab	33	265.500	0.268017	0.788686
Cov2Ex Ab and Cov2In Ab	21	77.000	1.338170	0.180842

*In* internal, road-facing trunk side, *Ex* external tree trunk side, *Cov* total moss cover, *Ab* abandoned road (Gryżów), *Bu* busy road (Lubrza), 0–0.2, 1–1.2, and 2–2.2 m

To discover the effect of road traffic abandonment, we compared the relevant plot series in the Lubrza and Gryżów tree hedgerows (Table 4). The *U* test revealed significant differences in all groups. The most important from a conservation view was the considerably higher moss cover on the inner side of tree trunks in abandoned road in Gryżów.

**Table 4:** The statistical results of Mann–Whitney *U* test comparing the moss cover between Lubrza and Gryżów plots at different heights

	*p* value	N Bu	N Ab
0ExBu and 0ExAb	0.000114	200	120
1ExBu and 1ExAb	0.000550	200	120
2ExBu and 2ExAb	0.002510	200	120
0InBu and 0InAb	0.000000	200	120
1InBu and 1InAb	0.000119	200	120
2InBu and 2InAb	0.001551	200	120

*In* internal, road-facing trunk side, *Ex* external tree trunk side, *Cov* total moss cover, *Ab* abandoned road (Gryżów), *Bu* busy road (Lubrza), 0–0.2, 1–1.2, and 2–2.2 m

**Fig. 4:**
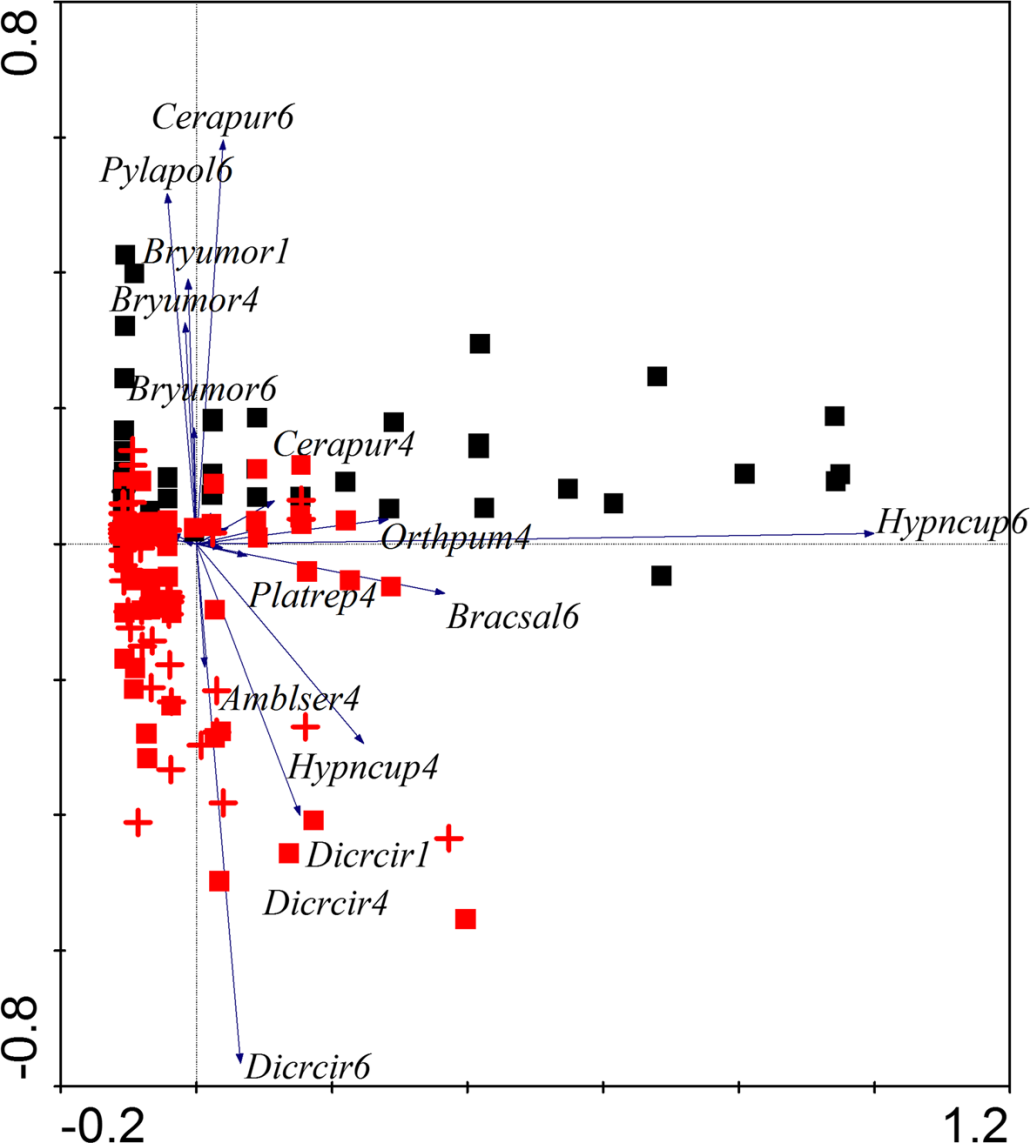
PCA scattered plot for samples from Lubrza external side (*black squares*) and Gryżów external side (*red squares*) and road-facing side (*red crosses*). Lubrza road-facing plots were excluded from the analyses because of data scarcity (only four samples with moss cover). *Cerapur4 Ceratodon purpureus* at 1.2-m height, *Cerapur6 Ceratodon purpureus* at 2.2-m height, *Pylapol6 Pylaisia polyantha* at 2.2-m height, *Bryumor1 Bryum moravicum* at 0.2-m height, *Bryumor4 B. moravicum* at 1.2-m height, *Bryumor6 B. moravicum* at 2.2-m height, *Orthpum4 Orthotrichum pumilum* at 1.2-m height, *Hypncup4 Hypnum cupressiforme* at 1.2-m height, *Hypncup6 Hypnum cupressiforme* at 2.2-m height, *Platrep4 Platygyrium repens* at 1.2-m height, *Bracsal6 Brachythecium salebrosum* at 2.2-m height, *Amblser4 Amblystegium serpens* at 1.2-m height, *Dicrcir1 Dicranoweisia cirrata* at 0.2-m height, *Dicrcir4 Dicranoweisia cirrata* at 1.2-m height, *Dicrcir6 Dicranoweisia cirrata* at 2.2-m height

The principal component analysis performed for the trees with moss cover *(N* = 229) as samples also revealed an apparent difference between the species structure of the Gryżów and Lubrza moss communities (black squares in Fig. 4). This was caused mainly by *Bryum moravicum*, *C. purpureus*, and *Pylaisia polyantha* being more abundant and frequent in busy road, and *D. cirrata*, *H. cupressiforme*, *Amblystegium serpens*, and *Platygyrium repens* being recorded mainly in Gryżów.

**Fig. 5:**
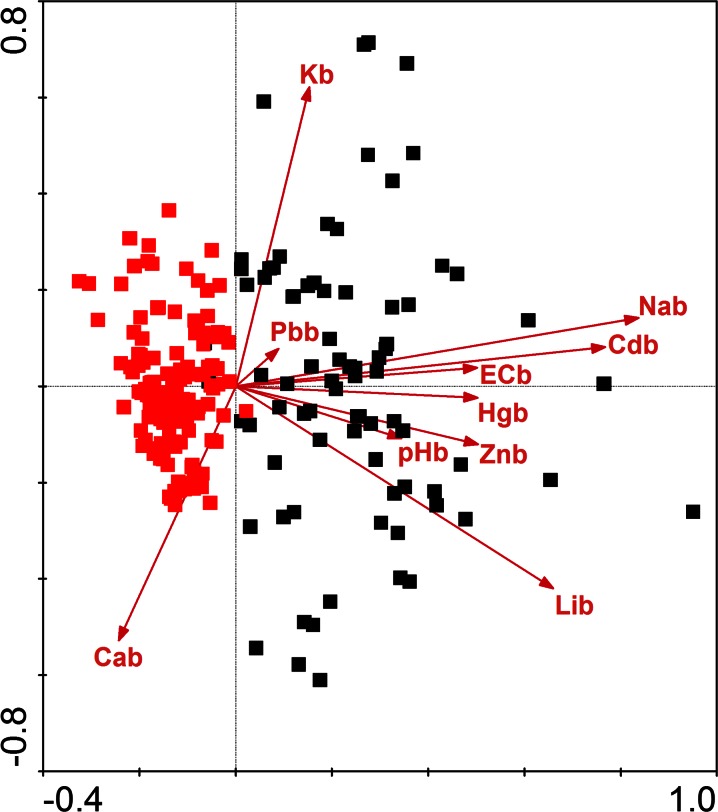
The redundancy analysis (RDA) for bryophyte plots in relation to bark contaminants. Abbreviations as in Table 1

**Fig. 6:**
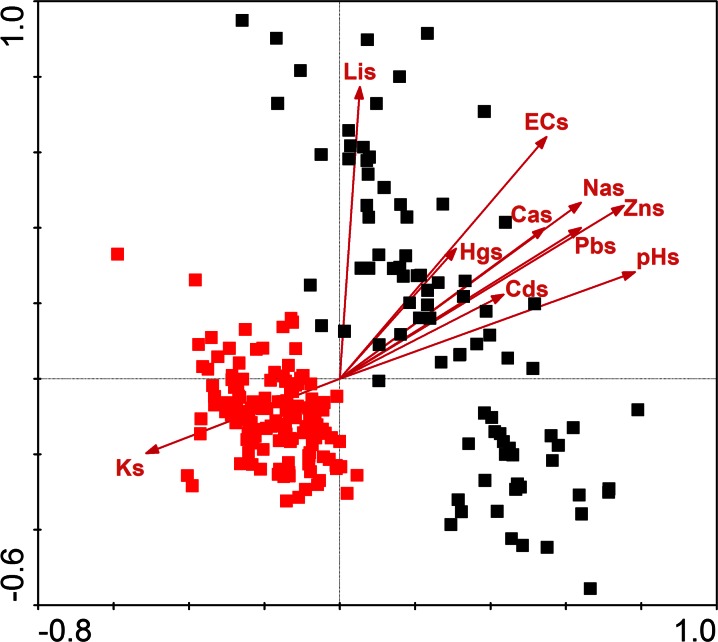
The RDA analysis of bryophyte plots in relation to soil contaminants. Abbreviations as in Table 1

Redundancy analysis clearly separated samples from the Gryżów and Lubrza allees (Figs. 5 and 6). Most of the contaminants, mainly toxic metals and elements responsible for salinity, had higher values in the Lubrza samples (soil and bark). Redundancy analysis of the soil pollutants showed that the most influential were reaction, zinc and lithium (for *F* statistics and significances values, see Table 2). In the bark, only the impact of sodium ions significantly explained the RDA model of bryophytes. The first two canonical axes explained 20.4 and 16.8 % of the sample variance, respectively). The raw data regarding the pollutant concentrations in bark and soils are given in Table 5.

**Table 5:** Environmental variables used in analyses: pollutant concentrations, soil reaction, and conductivity

	Outer side									Road facing side								
Busy road (Lubrza)																		
Cu*s* (mg/kg)	19.00	13.17	17.00	14.00	13.50	12.10	13.40	11.80	14.10	19.00	14.00	12.00	13.17	11.88	17.60	13.50	11.40	15.30
Zn*s* (mg/kg)	155.00	102.53	153.00	106.00	160.00	85.00	166.00	108.00	169.00	160.00	135.00	85.60	160.20	105.10	164.00	138.00	80.70	176.00
Pb*s* (mg/kg)	37.00	19.37	32.00	21.00	25.30	25.20	20.50	25.40	25.70	26.00	34.00	32.00	21.00	17.30	24.80	33.50	37.20	22.00
Cd*s* (mg/kg)	0.50	0.17	0.32	0.20	0.39	0.16	0.16	0.15	0.52	0.40	0.20	0.19	0.42	0.19	0.44	0.25	0.17	0.39
Hg*s* (mg/kg)	0.05	0.04	0.05	0.06	0.06	0.09	1.00	0.04	0.06	0.07	0.04	0.07	0.08	0.09	0.06	0.05	0.07	0.09
Na*s* (mg/kg)	880.00	620.00	894.00	625.00	916.00	696.00	632.00	672.00	898.00	880.00	665.00	695.60	878.00	711.00	893.00	645.00	678.00	889.00
K*s* (mg/kg)	914.00	893.30	911.00	899.00	902.00	885.00	961.00	841.00	935.00	885.00	876.00	741.00	913.50	882.00	894.00	847.00	771.00	908.00
Li*s* (mg/kg)	98.00	63.30	94.15	66.00	96.00	58.40	73.00	56.00	97.60	97.60	65.00	55.20	97.40	63.30	94.50	69.20	57.20	98.40
Ca*s* (mg/kg)	613.00	407.00	616.00	421.00	615.00	479.00	413.00	484.00	629.00	625.00	421.00	485.00	611.00	471.00	645.00	463.00	487.00	624.00
EC*s* (μS/cm)	131.00	259.20	137.00	268.00	227.00	146.00	265.50	271.00	149.00	133.50	281.00	145.70	301.00	157.00	135.00	295.00	187.00	330.00
pH*s*	6.00	6.04	5.72	6.98	6.06	6.18	5.93	6.06	6.19	5.93	6.04	6.21	6.04	6.20	5.78	6.16	6.25	5.98
Cu*b* (mg/kg)	34.00	17.95	36.00	18.20	17.95	7.82	17.95	10.15	16.40	33.13	17.95	7.82	17.95	7.82	35.50	18.40	6.88	19.80
Zn*b* (mg/kg)	669.00	269.80	681.00	272.00	658.00	125.00	259.00	68.00	674.00	675.00	274.00	22.16	668.20	274.00	714.00	285.50	34.60	652.00
Pb*b* (mg/kg)	81.00	28.02	83.00	31.00	79.00	9.30	80.60	29.20	10.50	80.80	28.80	9.30	81.00	28.90	82.40	30.80	11.40	85.50
Cd*b* (mg/kg)	0.63	0.50	0.71	0.80	0.59	0.64	0.69	0.68	0.58	0.65	0.61	0.58	0.52	0.60	0.64	0.58	0.55	0.59
Hg*b* (mg/kg)	0.10	0.05	0.15	0.09	0.09	0.03	0.08	0.06	0.09	0.06	0.08	0.06	0.04	0.05	0.07	0.08	0.05	0.06
Na*b* (mg/kg)	755.00	2,350.00	745.00	2,416.00	2,510.00	1,699.00	795.00	2,422.00	1,693.00	779.00	2,516.00	1,789.00	2,455.00	1,675.30	785.50	2,456.00	1,842.00	2,574.00
K*b* (mg/kg)	1,303.00	1,365.00	1,342.00	1,405.00	1,436.00	4,488.00	1,414.00	4,501.00	1,435.00	1,464.00	1,502.00	4,588.00	1,456.00	4,512.00	1,421.00	1,554.00	4,095.00	1,616.00
Li*b* (mg/kg)	62.00	109.00	66.00	119.00	118.40	88.20	86.40	117.90	86.20	71.00	118.50	85.90	119.80	88.20	74.50	126.00	88.40	122.50
Ca*b* (mg/kg)	8,314.00	14,850.00	8,271.00	14,905.00	8,313.00	14,825.00	14,520.00	8,216.00	8,264.00	8,245.00	14,652.00	14,235.00	8,313.00	8,210.00	8,105.00	13,950.00	14,255.00	9,564.00
EC*b* (μS/cm)	505.00	669.00	511.00	678.00	674.00	829.00	685.00	941.00	506.00	515.00	669.50	1,416.00	670.00	1,423.00	524.00	682.50	1,390.00	745.00
pH*b*	4.80	6.70	4.50	6.20	5.88	4.29	4.28	5.98	4.25	4.79	6.00	4.33	6.10	4.30	4.71	6.05	4.55	6.00
Abandoned road (Gryżów)																		
Cu*s* (mg/kg)	8.00	11.65	8.33	8.10	11.30	11.00	7.90	11.10	11.22	11.10	8.22	11.22	11.00	11.15	11.65	7.85	11.30	11.05
Zn*s* (mg/kg)	25.00	59.00	27.00	23.50	59.10	53.00	25.00	53.90	25.90	23.80	53.90	28.50	54.80	74.30	56.80	74.30	25.10	74.50
Pb*s* (mg/kg)	7.97	15.50	7.20	7.30	17.40	15.90	7.20	15.50	7.00	16.40	6.80	15.85	7.00	16.70	7.30	15.95	7.10	14.85
Cd*s* (mg/kg)	0.06	0.21	0.04	0.06	0.20	0.18	0.05	0.24	0.18	0.23	0.06	0.22	0.17	0.15	0.06	0.21	0.06	0.06
Hg*s* (mg/kg)	0.03	0.02	0.03	0.03	0.03	0.03	0.03	0.02	0.03	0.05	0.03	0.05	0.03	0.05	0.03	0.04	0.03	0.03
Na*s* (mg/kg)	512.60	505.00	503.00	487.00	497.00	500.00	519.00	378.00	509.00	501.00	458.00	500.00	465.00	476.00	471.50	492.00	468.00	510.00
K*s* (mg/kg)	981.00	944.00	967.00	964.00	119.00	999.00	816.00	782.00	766.00	926.00	997.00	999.00	927.00	989.00	936.00	990.00	957.00	1,001.00
Li*s* (mg/kg)	65.00	68.00	63.60	65.20	65.90	61.10	67.00	65.80	66.00	65.00	68.00	57.50	67.00	64.00	63.80	55.80	65.40	65.90
Ca*s* (mg/kg)	335.00	376.00	340.00	337.00	361.00	367.00	327.00	440.00	333.00	364.00	419.00	372.00	337.00	333.50	335.00	362.00	359.00	428.00
EC*s* (μS/cm)	49.70	71.00	68.20	70.20	45.30	48.20	69.10	69.30	44.80	45.30	65.80	70.90	69.10	70.40	72.60	44.30	67.50	48.20
pH*s*	5.44	5.33	5.47	5.46	5.28	5.25	5.41	5.44	5.04	5.28	5.06	5.55	5.48	5.35	5.02	5.28	5.01	5.33
Cu*b* (mg/kg)	12.20	10.00	11.97	13.40	8.85	10.40	11.90	5.80	9.69	12.33	9.69	13.20	11.60	9.50	12.43	5.80	5.70	9.45
Zn*b* (mg/kg)	33.00	40.00	35.10	36.00	41.20	35.80	32.80	34.80	17.40	36.00	35.40	17.20	46.80	35.10	18.10	36.40	33.00	18.00
Pb*b* (mg/kg)	46.00	16.30	44.60	47.80	15.80	16.58	49.39	45.90	13.80	47.80	46.40	12.80	16.60	14.10	15.70	16.50	16.60	12.80
Cd*b* (mg/kg)	0.41	0.42	0.42	0.41	0.41	0.40	0.45	0.46	0.44	0.41	0.42	0.39	0.38	0.44	0.47	0.50	0.40	0.48
Hg*b* (mg/kg)	0.05	0.03	0.06	0.06	0.03	0.03	0.06	0.04	0.03	0.03	0.04	0.03	0.04	0.06	0.02	0.03	0.06	0.06
Na*b* (mg/kg)	178.00	109.00	183.00	187.30	106.00	98.00	182.30	548.00	105.00	178.00	109.00	174.00	187.30	179.00	566.00	109.00	171.00	102.00
K*b* (mg/kg)	1,145.80	1,002.00	1,144.00	1,165.00	1,003.00	1,016.00	1,128.00	4,002.00	1,013.00	1,144.00	1,016.00	1,128.00	1,140.00	998.00	1,139.00	4,017.00	4,030.00	1,036.00
Li*b* (mg/kg)	68.00	85.30	64.30	62.60	66.40	67.00	60.90	64.30	82.10	65.00	68.00	69.40	84.00	64.30	82.10	66.50	65.70	65.50
Ca*b* (mg/kg)	12,561.00	13,776.00	12,895.00	12,675.00	14,523.00	13,985.00	12,610.00	13,341.00	11,985.00	12,561.00	13,776.00	12,855.00	13,890.00	12,345.00	12,244.00	12,287.00	11,854.00	13,587.00
EC*b* (μS/cm)	322.00	335.00	319.00	318.00	354.00	354.00	335.00	352.00	342.00	325.00	354.00	335.00	344.00	329.00	322.00	358.00	346.00	324.00
pH*b*	4.58	5.10	4.30	4.26	4.38	4.56	4.25	5.18	5.10	4.30	4.56	4.30	5.18	4.44	5.16	5.18	5.15	4.26

## Discussion

In comparison to the bryoflora of urban or rural areas of Europe, the observed moss richness on the investigated tree plots, especially those in abandoned road in Gryżów, was relatively high (Gilbert 1968; Loppi et al. 1999; Zechmeister et al. 2003; Larsen et al. 2007; Smith et al. 2010). Obviously, if compared to forested areas, even anthropogenic tree plantations, the epiphytic moss diversity on veteran trees in the agricultural landscape of Opole Province would be considerably lower (Lesica et al. 1991; Boch et al. 2013). The same holds true for the tropical bryophyte flora of tree trunks (e.g., Roberts et al. 2005; Gradstein and Culmsee 2010) or lichen communities (Lesica et al. 1991; Larsen et al. 2007; Ódor et al. 2013).

Avenues of trees along roads are very often colonized by epiphytic bryophytes. Air flows made by traffic significantly contribute to the spreading of the spores and the gemmae of the mosses and liverworts. In particular, invasive or expansive species can spread easily by this corridor. This has been shown in the studies concerning the spreading of the expansive moss species *Orthodicranum tauricum* and *D. cirrata* (Plášek 2001a, b; Stebel and Plášek 2001; Stebel et al. 2012). Both of the expansive species were also recorded in the studied allees in Lubrza and Gryżów. Bryophytes that reproduce strictly by gemmae distinctly exploit this way of migration. The asexually produced multicellular bodies that always weigh more than spores would be poorly distributed by wind. Examples of such species found in our study are the mosses *Tortula papillosa* and *Bryum moravicum*.

In addition to the expansive taxa discussed above, most commonly distributed obligatory and facultative epiphytic species of bryophytes were recorded in the study areas. Obligate epiphytes grow mainly at height of 50–60 cm and more above the ground. Although some obligate epiphytes are occasionally recorded growing on man-made substrates such as concrete, in most cases, they grow on the bark of deciduous trees. Such obligate epiphytes are represented by species from the genera *Orthotrichum* and *Nyholmiella*. Both of the genera regularly occur on trees along roads. In the study areas, three epiphytic species from *Orthotrichum* were found. However, the common species *Nyholmiella obtusifolia* was surprisingly not observed here (cf. Sawicki et al. 2011).

The bases of tree trunks are characterized by the occurrence of a high number of facultative epiphytic bryophytes. These species grow mostly on soil, rocks, and concrete, or their substrate preferences are very vague. The fact that they often occur on tree bases and buttress roots is mainly due to the mild microclimate conditions created by the surrounding vegetation. In addition, rain-displaced soil can splatter around and cover the base of trees in a thin layer. This distinctly changes the substrate character of this part of trees. The large group of facultative epiphytic bryophytes is a very heterogeneous group, including species with ecological optima on different substrata, a feature likely related to different degrees of tolerance to drought. The most commonly recorded species in the studied areas included *C. purpureus*, *Brachythecium* sp. div., *H. cupressiforme*, and *Amblystegium serpens*.

The proportional representation of obligate and facultative epiphytes in the allees was different. While the impact of transport in busy road in Lubrza appreciable changed the species spectrum of bryophytes toward facultative epiphytes, in Gryżów, the proportion of obligate and facultative epiphytes was rather balanced. The almost 30 years during which the avenue near Gryżów was not exposed to the strong influence of pollution by exhaust gases, dust, and salting helped to create rich bryophyte communities, especially of obligate epiphytes. The population of obligate epiphytic bryophytes was significantly larger there than in Lubrza. However, both the number of facultative epiphytic species and their population size were significantly larger in Lubrza.

Synanthropic species occupy a special position within facultative epiphytes. They are often very resistant to pollution by exhaust gases, as well as salt, oil, and some toxic substances. Such species are represented in the study areas by *C. purpureus*, *Barbula unguiculata*, and *Tortula muralis*. These species often grow on the bases of trees covered in dust. In the same polluted conditions, they can also be found growing on the ground among the trees. It is commonly known also for other groups of indicator organism that some of them could tolerate considerably high concentrations of toxic substances. They are often named as urbanophilous species. Examples are known from urbanized areas of Great Britain, Sweden, or France where *Phaeophyscia orbicularis*, *Anaptychia ciliaris*, *Lecanora conizaeoides*, or *Xanthoria polycarpa* occur in city central parts with extreme concentrations of nitrogen oxides and heavy dust burdens (Gombert et al. 2004; Hultengren et al. 2004; Larsen et al. 2007).

There were very divergent results for the occurrence of epiphytic bryophytes on the sides of trees that were facing or opposite to the public road. In abandoned road in Gryżów, the differences in cover between the sides were negligible after almost 30 years without vehicles, while in Lubrza, the bryophytes were recorded as growing almost exclusively on the opposite side of trees from the road. No epiphytic species of moss or liverwort was recorded on the road-facing side in the area. This shows the clear consequence of long-term pollution, which eliminated even the otherwise resistant synanthropic species from the side facing the road.

Although bryophytes are commonly classified in the biomonitoring of airborne pollutants as relatively resistant plants (e.g., Castello 1996; Bignal et al. 2008; Carvajal et al. 2010), our study shows that road traffic could have a significant influence on moss diversity and bryophyte assemblages. The species composition with respect to structure, abundances, and synanthropization reflects the environmental pollution by toxic metals and salinity. We found that the contaminants most influencing bryophyte diversity were lithium, zinc, general soil reaction, and the sodium ion concentration, which is closely related to salinity. This is in line with recent studies in London where bark acidity significantly influenced moss and lichen richness (Larsen et al. 2007). However, in our case, the acidity was slightly lower in abandoned road in Gryżów, where the bryoflora was considerably more diverse. This could be because in rural areas with consistent environmental acidity, the pH reaction of bark or soil has no crucial effect on moss richness within a small range of values (ca. pH 4.7–6.0). The redundancy analysis also showed that other physicochemical compounds of soil and bark could affect moss diversity, however without statistical significance (e.g., electroconductivity, Hg, Cd, Pb). Apparently, toxic metal deposits also affect the growth, physiology, enzyme activity, chemistry, and finally senescence of plants (Bignal et al. 2004). However, investigations have shown that heavy metal uptake and retention efficiencies differ from species to species according to morphological and physiological variables (e.g., Herpin et al. 1996).

The study shows that the implementation of a bypass road can be an effective conservation tool for moss flora protection. It is important to implement it at least in rural areas where relevant space for bypass roads is available. This could raise the effectiveness of floral diversity conservation, as has been frequently postulated (e.g., Sabovljević et al. 2001; Nowak and Nowak 2004b). Even if the new road is not very far from the conservation object (in our case, the monumental tree allee), the airborne pollutions are significantly diminished and the microhabitat and environmental parameters could return relatively rapidly to previous conditions. That helps not only the trees and their health but also allows the whole biocoenosis related to hedgerows to recover, including moss diversity. Even in a relatively short time, the bryophytes could gain considerably higher richness and diversity in comparison to trees that are still exposed to road traffic contamination. Thus, road traffic abandonment helps not only synanthropic mosses but also obligatory epiphytes and species regarded as rare or threatened in the area such as *Tortula papillosa* which is in the last decades, severely declining taxon (Kučera and Váňa 2003).
